# Low serum sphingolipids in children with attention deficit-hyperactivity disorder

**DOI:** 10.3389/fnins.2015.00300

**Published:** 2015-08-25

**Authors:** Marcela P. Henríquez-Henríquez, Sandra Solari, Teresa Quiroga, Benjamin I. Kim, Richard J. Deckelbaum, Tilla S. Worgall

**Affiliations:** ^1^Department of Pediatrics, Institute of Human Nutrition, College of Physicians and Surgeons, Columbia UniversityNew York, NY, USA; ^2^Department of Clinical Laboratories, School of Medicine, Pontificia Universidad Católica de ChileSantiago, Chile; ^3^Department of Pathology and Cell Biology, Columbia UniversityNew York, NY, USA

**Keywords:** ADHD, sphingolipids, sphingomyelins, ceramides, biomarker, endophenotype

## Abstract

**Background:** Attention deficit-hyperactivity disorder (ADHD) is the most prevalent neuropsychiatric condition in childhood. ADHD is a multifactorial trait with a strong genetic component. One neurodevelopmental hypothesis is that ADHD is associated with a lag in brain maturation. Sphingolipids are essential for brain development and neuronal functioning, but their role in ADHD pathogenesis is unexplored. We hypothesized that serum sphingolipid levels distinguish ADHD patients from unaffected subjects.

**Methods:** We characterized serum sphingolipid profiles of ADHD patients and two control groups: non-affected relatives and non-affected subjects without a family history of ADHD. Sphingolipids were measured by LC-MS/MS in 77 participants (28 ADHD patients, 28 related controls, and 21 unrelated controls). ADHD diagnosis was based on the Diagnostic and Statistical Manual of Mental Disorders (DSM IV-TR). Diagnostic criteria were assessed by two independent observers. Groups were compared by parametrical statistics.

**Results:** Serum sphingomyelins C16:0, C18:0, C18:1, C24:1, ceramide C24:0, and deoxy-ceramide C24:1 were significantly decreased in ADHD patients at 20–30% relative reductions. In our sample, decreased serum sphingomyelin levels distinguished ADHD patients with 79% sensitivity and 78% specificity.

**Conclusions:** Our results showed lower levels of all major serum sphingomyelins in ADHD. These findings may reflect brain maturation and affect neuro-functional pathways characteristic for ADHD.

## Introduction

Attention deficit-hyperactivity disorder (ADHD) is a neurobehavioral condition characterized by persistent, cross-situational and developmentally inappropriate levels of inattention, hyperactivity, and impulsiveness (American Psychiatric, 2000). ADHD affects 8–12% of children of school age and is thus the most common pediatric neuropsychiatric disorder (Polanczyk et al., 2014). ADHD is considered a multifactorial trait in which genetic factors account for about 75–80% of the phenotypic variance and environmental factors, such as premature delivery, perinatal hypoxia, maternal smoking, alcohol consumption, and others, may contribute to the remaining 20–25% (Thapar et al., 2013; Li et al., 2014; Polanczyk et al., 2014). The therapeutic effect of stimulants has led to wide acceptance of the dopaminergic hypothesis, a dysfunction in catecholaminergic/dopaminergic transmission, as a core mechanism for the disorder (Swanson et al., 2000; Tripp and Wickens, 2009), although associations identified between ADHD and monoaminergic gene variants account for a very modest risk effect (Li et al., 2014). A recent genome-wide association study suggested that exploration of alternative/complementary etiological factors related to neuritic outgrowth and maturation should be considered (Bralten et al., 2013). This is in line with neuroimaging studies, which increasingly suggest white/gray matter anomalies in prefrontal cortex, temporo-parietal regions, striatum, and cerebellum in ADHD patients (Casey et al., 2007; Silk et al., 2009; Helpern et al., 2011; Nagel et al., 2011; Peterson et al., 2011; de Zeeuw et al., 2012; Cortese et al., 2013; Greven et al., 2015). In this context, a prominent neurodevelopmental hypothesis proposes that ADHD involves a lag in brain maturation (Castellanos et al., 2002; Rubia, 2007; Shaw et al., 2007).

Sphingolipids, highly abundant in nervous tissue, affect neuronal and glial proliferation, differentiation, apoptosis as well as membrane permeability to Ca^2+^ and K^+^, relevant to the generation and propagation of the nervous impulse and neurotransmitter release (Colombaioni and Garcia-Gil, 2004; Gielen et al., 2006; Posse De Chaves and Sipione, 2010). Cell and animal models underscore the key function of sphingolipids in neurite growth and mass of cerebellum and forebrain (Hirabayashi and Furuya, 2008; Imgrund et al., 2009; Ginkel et al., 2012). Deficiency of ceramide synthase-2 that generates sphingolipids with C22–C24 fatty acyl chains, results in 50% loss of compacted myelin and 80% loss of CNS myelin basic protein (Imgrund et al., 2009). Similarly, a 60% reduction of myelin-associated glycoprotein in cerebellum and forebrain characterizes mice deficient in ceramide synthase-1, the enzyme that generates C18:0 sphingolipids. Hyperactive behavior characterizes mice deficient of ceramide synthase -6 that generates C16:0 sphingolipids (Ebel et al., 2013). In premature infants, supplementation of milk with sphingomyelins has been associated with significant improvements in attention and memory measurements at 18 months, suggesting positive effects on neurobehavioral development (Tanaka et al., 2013). Based on these observations and reported correlations between serum and CSF sphingolipids (Mielke et al., 2010, 2011) we hypothesized that serum sphingolipid levels distinguish ADHD patients from unaffected subjects. The exploration of sphingolipids as potential pathogenic factors and/or biomarkers for ADHD is new.

## Methods

### Participants

This case-control study was designed to compare serum sphingolipid profiles of ADHD patients with two independent control groups: unaffected first degree relatives of ADHD patients, sharing housing, and dietary patterns with the index cases and unaffected subjects with no family history of ADHD. We studied 77 subjects belonging to one of the following 3 groups: (1) ADHD patients (*n* = 28; 14 males), (2) unaffected first-degree relatives of ADHD patients [mother, father, or siblings of the index cases included in group (1), *n* = 28; 4 males], and (3) unaffected participants without a family history of ADHD (also referred to as unaffected unrelated subjects or non-related controls, *n* = 21; 8 males). Table 1 summarizes relevant characteristics of the three groups. ADHD diagnosis was based on the criteria of the Diagnostic and Statistical Manual of Mental Disorders in all cases (DSM IV-TR) (American Psychiatric, 2000). Criteria were assessed by at least two independent observers (most commonly: parents and teachers), according to current guidelines (Seixas et al., 2012; Hauk, 2013). All participants were recruited from an ongoing study exploring the role of polyunsaturated fatty acids in ADHD pathogenesis (Henríquez-Henríquez et al., 2012). Participants with clinical evidence/anamnestic antecedent of major systemic and neurological illness and taking ω-3/ω-6 supplementation were excluded from the study.

**Table 1:** **Relevant clinical and epidemiological characteristics in the three groups studied**.

		**Non-related controls**	**Related controls**	**ADHD patients**
Number of subjects		21	28	28
Age (years)	25th percentile	12.3	23.6	11
	Median	23.5	34	12.8
	75th percentile	34.8	43	17
Gender	Male	8	4	14
	Female	13	24	14
Number of DSM IV-TR positive criteria	Inattention	1 (0–3)	1 (0–5)	7 (4–9)
	Hyperactivity/Impulsivity	1 (0–3)	1 (0–5)	6 (0–9)
ADHD subtype	Predominantly hyperactive-impulsive	N/A	N/A	1
	Predominantly inattentive	N/A	N/A	10
	Combined type	N/A	N/A	17
Treatment	D-Amphetamine	N/A	N/A	7
	Methylphenidate	N/A	N/A	21

Age is described in terms of the percentiles 25th, 50th, and 75th due to non-Gaussian distribution of this variable inside the groups.

ADHD patients were between 5 and 18 years old (median = 12.7 years; 25th percentile = 11 years; 75th percentile = 17 years) and referred from general psychiatric/neurological and family medicine outpatient clinics serving a medium income area of urban Santiago, Chile. Seventeen patients were clinically classified as ADHD combined subtype, 10 patients as predominantly inattentive, and one patient as predominantly hyperactive-impulsive (American Psychiatric, 2000). This distribution agrees with the subtype prevalence generally described for clinically referred ADHD patients across the globe (Willcutt, 2012). All patients were treated with either d-amphetamine or methylphenidate in doses ranging 10–30 mg/day. A medication free washout period of 24 h was required prior to blood analyses (t1/2 for methylphenidate at 10–20 mg/kg: 2–4 h; t1/2 for d-amphetamine 6–12 h).

ADHD diagnosis was excluded from the unaffected groups (groups 2 and 3) by means of a brief clinical interview exploring DSM-IV-TR criteria (American Psychiatric, 2000). We required that unaffected relatives participating in this study were living and eating in the same home with the ADHD index case, to control dietary and other unknown environmental factors. Group 3 (consisting of unaffected participants without a family history of ADHD) were children recruited from a medium-income school in the same urban area and healthy adults recruited from the same family medicine services at the time of general annual checkup. Family history of ADHD was anamnestically explored in group 3 considering first and second degree relatives. Ages of unaffected relatives (group 2) ranged from 10 to 64 years (median = 34 years; 25th percentile = 23.6 years; 75th percentile = 43 years). In group 3 (non-related controls) ages ranged from 7 to 51 years (median = 23.5 years; 25th percentile = 12.3 years; 75th percentile = 35.6 years). Because age and gender distributions significantly differ among the 3 groups (*p* = 0.01 and *p* = 0.001, respectively), we controlled for those in all subsequent statistical analyses.

All procedures performed as part of this study have been approved by the Ethics Committee of Pontificia Universidad Católica de Chile. The study was fully explained to children and their parents, and they both agreed to participate by signing written consent and assent forms.

### Serum sphingolipid analysis

Blood samples were obtained after an 8 h fast. Samples were centrifuged and obtained serum was stored at −20°C until analysis.

**Figure 1:**
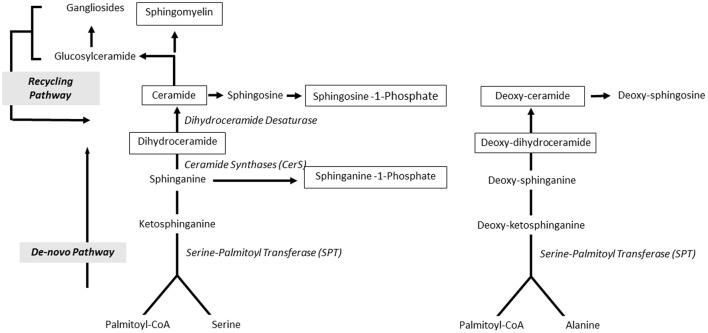
**Sphingolipid metabolic pathways**. Ceramide is central in sphingolipid metabolism, produced by *de novo* and recycling pathways. Serine and palmitoyl-CoA are substrates of serine palmitoyl-transferase (SPT), the rate limiting enzyme of *de novo* synthesis that generates ketosphinganine from serine and palmitoyl-CoA (bottom). Ketosphinganine is reduced to form sphinganine that is N-acylated by ceramide synthases with fatty acids of different chain lengths to form dihydroceramides. Dihydroceramides are desaturated by dihydroceramide desaturase (DES) to generate ceramides. Ceramides can be metabolized to sphingomyelin or glycosphingolipids. SPT generates deoxyceramides when utilizing alanine instead of serine. Notably, deoxyceramides only originate from *de novo* synthesis. The recycling pathway generates ceramides from sphingomyelin and other complex sphingolipids. Ceramidases degrade ceramides and release sphingoid bases, which are reutilized for complex sphingolipid biosynthesis or phosphorylated to sphingosine-1-phosphate. Boxed compounds indicate molecular species assayed in this study.

Sphingolipid profiles include the following molecular species: (1) sphinganine-1-phosphate and sphingosine-1-phosphate; (2) ceramides C16:0, C18:0, C20:0, C22:0, C24:0, and C24:1; dihydroceramides C18, C18:1, C24:0, and C24:1; deoxy-ceramide C16:0 and C24:1 and deoxy-dihydroceramides C16:0 and C24:1; (3) sphingomyelins (SM) SM C16:0, SM C18:0, SM C18:1, and SM C24:1. Their relative location in the sphingolipid metabolic pathway is summarized in Figure 1. Sphingolipid levels were quantified by high performance liquid chromatography-triple quadrupole-tandem mass spectrometry (HPLC-MS/MS) on an Agilent 1200 HPLC system, equipped with an Agilent C18 column as reported (Bui et al., 2012). Briefly, sphingolipids were extracted overnight in a 1:30 v/v solution of diethylamide 10%/dichloromethanol: methanol 1:1, at room temperature using sphingomyelin C12 (0.120 μM) as internal standard. HPLC conditions were: Mobile phase A was methanol/water/chloroform/formic acid (55:40:5:0.4 v/v); Mobile phase B was methanol/acetonitrile/chloroform/formic acid (48:48:4:0.4 v/v). The chromatography column was first pre-equilibrated for 6 s. After this period, the gradient gradually increased to 60% mobile phase B and 100% mobile phase B. 100% mobile phase B was held for 1.9 min. Flow rate was 0.6 ml/min. Injection into the HPLC-MS/MS system was set at 3 μl. All measurements were performed in triplicates.

### Data analysis

The final serum level for each patient and analyte was calculated by averaging triplicate measurements. Subsequent statistical analyses were performed on these final levels. Average inter-assay imprecision for sphingolipid quantification was 8.7% (ranging from 2.5% for sphingomyelins to 12.3% for deoxyceramides).

### Statistical analysis

The normal distribution of individual sphingolipids was evaluated by the Kolmogorov-Smirnov test after outlier detection/rejection by the Tukey method (Tukey, 1977). All sphingolipids were normally distributed. Group differences were examined by general linear models (GLM) using sphingolipid levels as the dependent variable and “clinical group,” “age,” and “gender” as covariates. “Clinical group” was defined as a categorical variable with 3 levels: ADHD patients, unaffected relatives of ADHD patients and unaffected unrelated subjects. *Post-hoc* pair-wise comparisons were performed when significant effect for the variable “clinical group” was detected and significance was maintained as *p* = 0.05. This approach was verified using the adaptive procedure described by Benjamini and Hochberg (BH-A) for controlling the False Discovery Rate (FDR) (Kromrey and Hogarty, 2002), with the same results in terms of the rejection of the null hypothesis. All analyses were conducted using SAS 9.2 software (SAS Institute, Cary, North Carolina).

To evaluate the ability of the assayed sphingolipids for correctly classifying ADHD affected and non-affected subjects, we performed Receiver Operating Characteristic (ROC) analyses (Fawcett, 2004), using the method of Delong et al for the calculation of the standard error of the area under the curve (DeLong et al., 1988).

## Results

### Analysis of sphingomyelins

**Figure 2:**
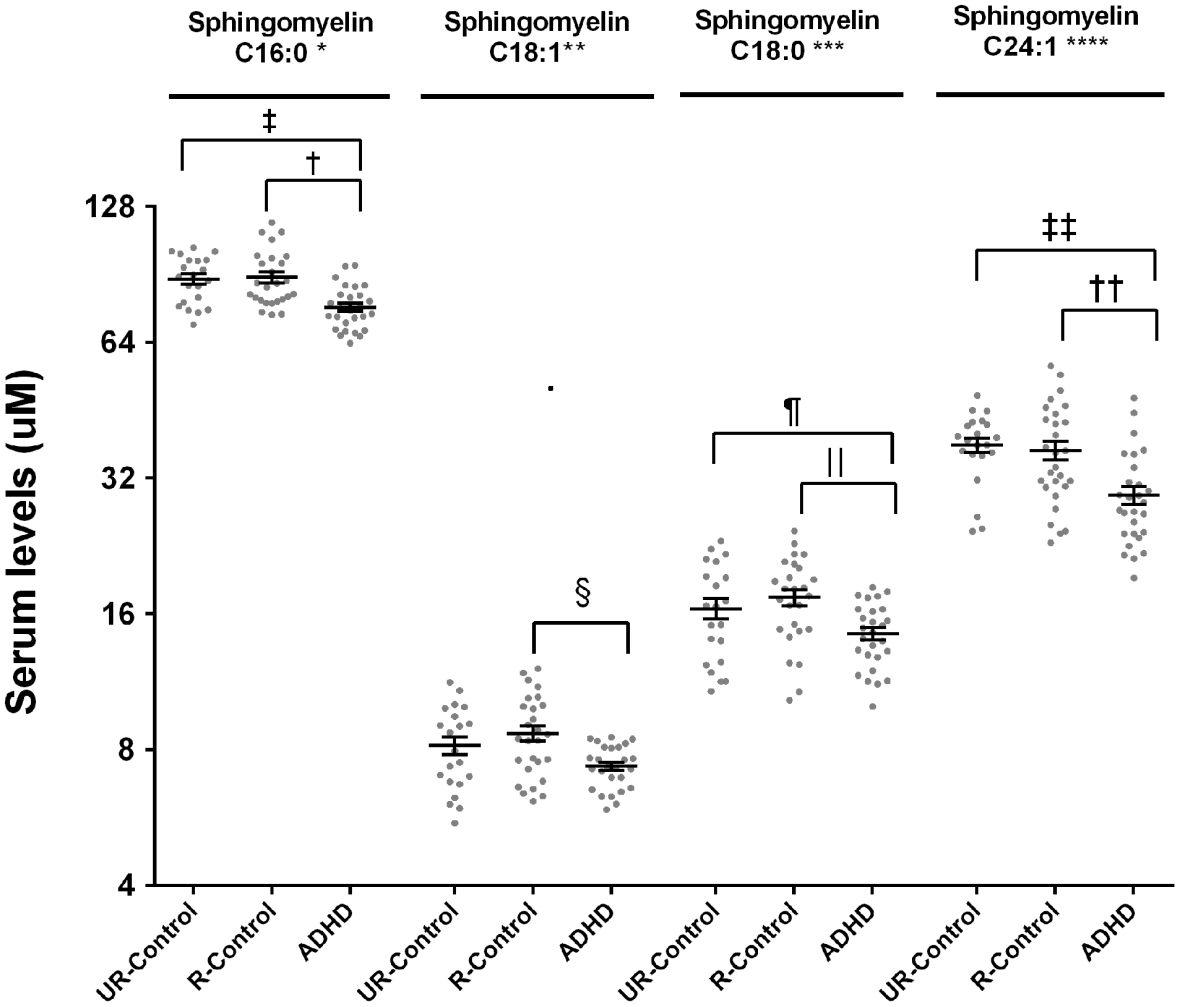
**Serum levels for sphingomyelins in ADHD patients, their unaffected relatives and unaffected subjects without family history of ADHD**. Serum sphingomyelin levels were measured by LC-MS/MS as described. Results are expressed in μM. Plots represent individual values for all data included in the analysis. Mean and SEM are indicated by bars. UR-Controls, Unaffected subjects without family history of ADHD; R-Controls, Unaffected relatives of ADHD patients. ^*^p = 0.0002, ^**^p = 0.05, ^***^p = 0.014, ^****^p = 0.0014, ^†^*p* = 0.0005, ^‡^*p* = 0.0003, ^§^*p* = 0.01, ^‖^*p* = 0.0045, ^¶^*p* = 0.046, ^††^*p* = 0.013, and ^‡‡^*p* = 0.0004.

ADHD patients had lower serum levels of all assayed SM species. The “clinical group” effect was significant in all species after controlling by age and gender (SM C16:0 *p* < 0.0001; SM C18:1 *p* = 0.05; SM C18:0 *p* = 0.01; SM 24:1 *p* = 0.001) (Figure 2). Pairwise comparisons confirmed significant differences between ADHD patients and both control groups for all the species, except for sphingomyelin C18:1, where ADHD patients were significantly different only when compared to related controls. The biggest relative difference was observed for sphingomyelin C24:1, which was 21% lower in ADHD patients. Relative differences in other species ranged from 13% (SM C16:0) to 18% (SM C18:0).

### Analysis of ceramides, dihydroceramides, and deoxy-ceramides

**Figure 3:**
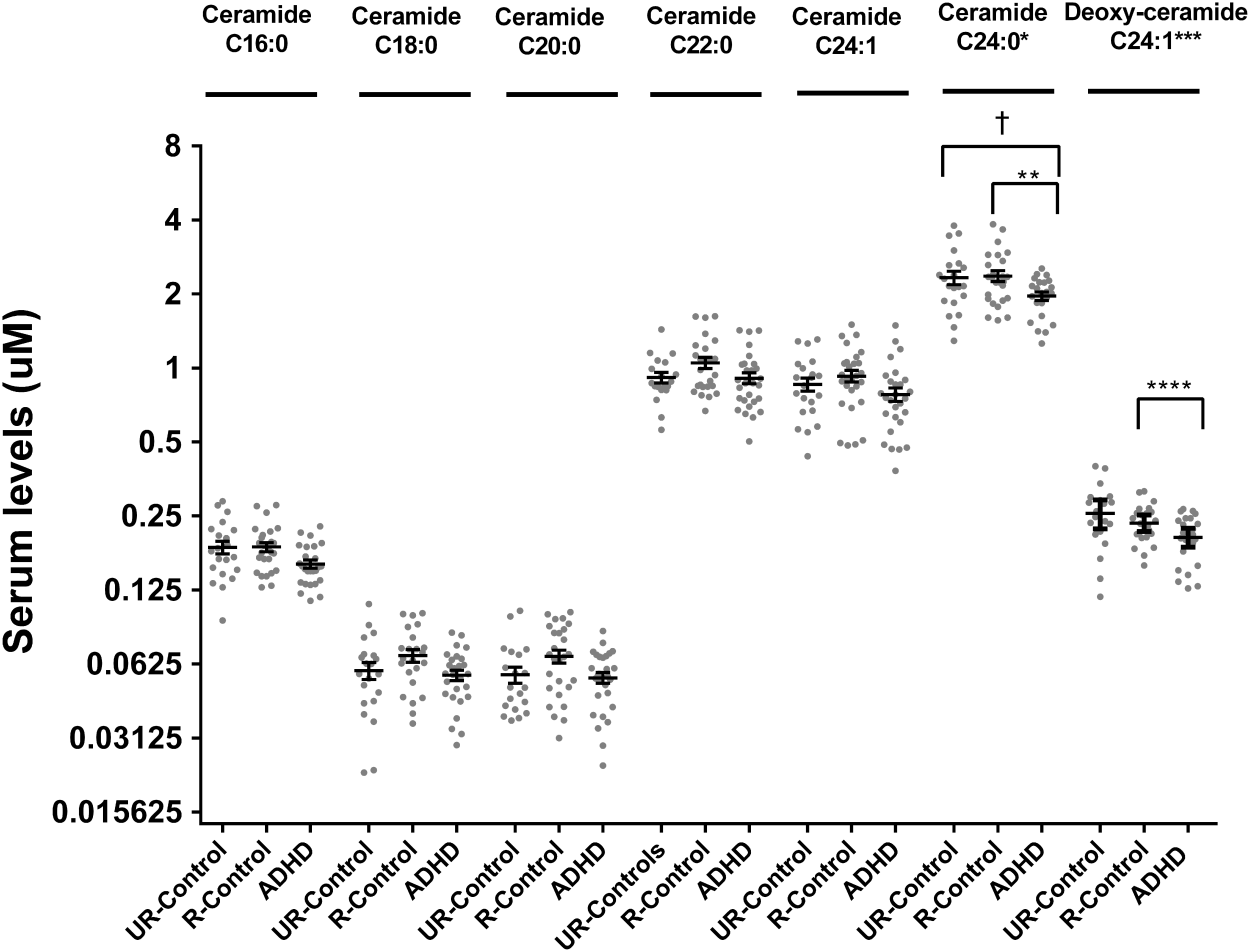
**Serum levels for ceramides and deoxy-ceramides in ADHD patients, their unaffected relatives and unaffected subjects without family history of ADHD**. Serum ceramide levels were measured by LC-MS/MS as described. Results are expressed in μM. Plots represent individual values for all data included in the analysis. Mean and SEM are indicated by bars. UR-Controls, Unaffected subjects without family history of ADHD; R-Controls, Unaffected relatives of ADHD patients. ^*^p = 0.03, ^**^p = 0.018, ^***^p = 0.003, ^****^p = 0.01, and ^†^*p* = 0.0411.

Analysis of ceramides (C16:0, C18:0, C22:0, C24:1, and C24:0) showed 20% lower ceramide C24:0 in ADHD patients compared to unaffected relatives (*post-hoc* pair-wise comparison *p* = 0.02) (Figure 3). This difference was maintained when comparing ADHD patients with non-related controls (*post-hoc* pair-wise comparison *p* = 0.04). We explored dihydroceramide levels to evaluate if decreased ceramide C24:0 could originate from decreased *de novo* synthesis and found no differences between groups (data not shown). To further evaluate the *de-novo* pathway we also measured deoxy-ceramides, which originate obligatory through the *de-novo* pathway (Figure 1). We measured the four most abundant species for which external standards are available (deoxy-dihydroceramides C16:0, C24:1, and deoxy-ceramides C16:0, C24:1) and found deoxy-ceramide C24:1 to be 30% lower in ADHD patients compared to related controls (pairwise comparison *p* = 0.003; *p*-value “clinical group” effect in GLM = 0.01) (Figure 3). A similar, but not significant trend was observed when comparing deoxy-ceramide C24:1 in ADHD patients to levels found in non-related controls (relative difference = 14%; *post-hoc* pairwise comparison *p* = 0.1).

### Sphingosine-1-phosphate and sphinganine-1-phosphate levels

We evaluated levels of sphingosine-1-phosphate and sphinganine-1-phosphate among the three groups, of potential interest as sphingosine-1-phosphate has been linked to regulation of neuronal excitability and neurotransmitter release (Colombaioni and Garcia-Gil, 2004). Mean serum sphingosine-1-phosphate was 1.55 μM (SD = 0.38 μM) in ADHD patients. Concentrations did not differ (*p* = 0.48) from those found in unaffected relatives (mean ± SD = 1.44 ± 0.38 μM) or unaffected controls without a family history of ADHD (mean ± SD = 1.59 ± 0.38 μM). Mean serum sphinganine-1-phosphate was 0.23 μM (SD = 0.008 μM) in ADHD patients, 0.2 μM (SD = 0.01 μM) in unaffected relatives and 0.22 μM (SD = 0.007 μM) in non-related controls. Sphinganine-1-phosphate concentrations were not different between groups (*p* = 0.27). These findings suggest that serum sphingoid bases are not different in ADHD patients.

### Low serum sphingolipids distinguish ADHD patients from unaffected subjects

**Figure 4:**
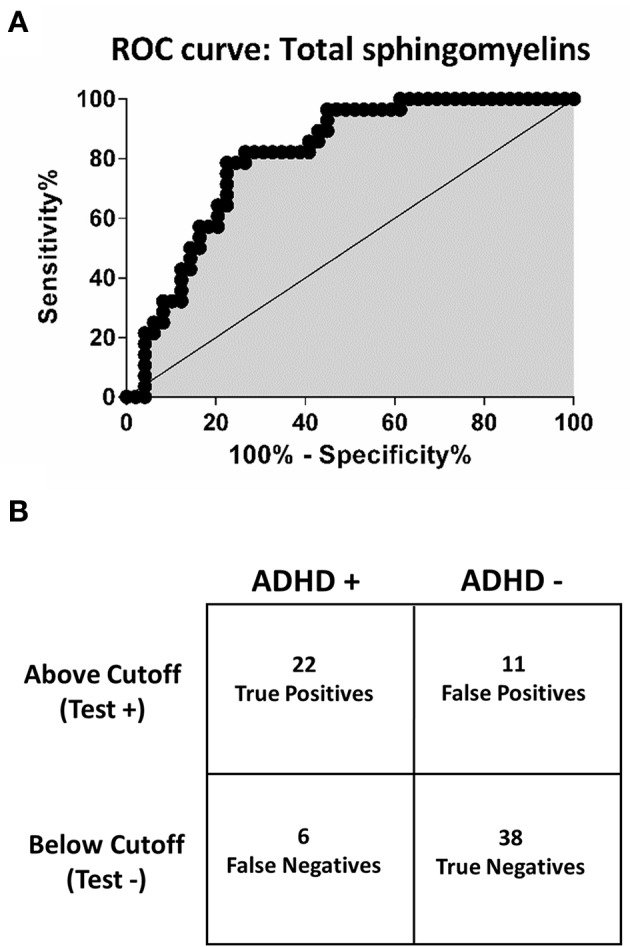
**Receiver operating characteristic (ROC) curve and confusion matrix for total sphingomyelins. (A)** Receiver operating characteristic curve (ROC). The point of best discrimination was determined to be 134 uM total sphingomyelins. Total sphingomyelins refers to all assayed sphingomyelins. **(B)** Contingency table (confusion matrix) for total sphingomyelins. Total sphingomyelins < 134 μM provide a sensitivity of 79% and specificity of 78%. Considering a prevalence of 10% for ADHD, the negative predictive value for total sphingomyelins is 97%.

We built Receiver Operating Characteristic (ROC) curves to evaluate whether any of the assayed sphingolipids would discriminate between ADHD-affected and unaffected subjects with adequate levels of sensitivity and specificity. The best detection accuracy was obtained for total SM levels (the sum of all detected SM), with an AUC of 0.81 (CI: 0.71 to 0.89; *p* < 0.0001), 79% sensitivity and 78% specificity (Figure 4). The estimated negative predictive value, considering 10% ADHD prevalence, was 97%.

### Sphingolipids as potential endophenotypes for ADHD

**Figure 5:**
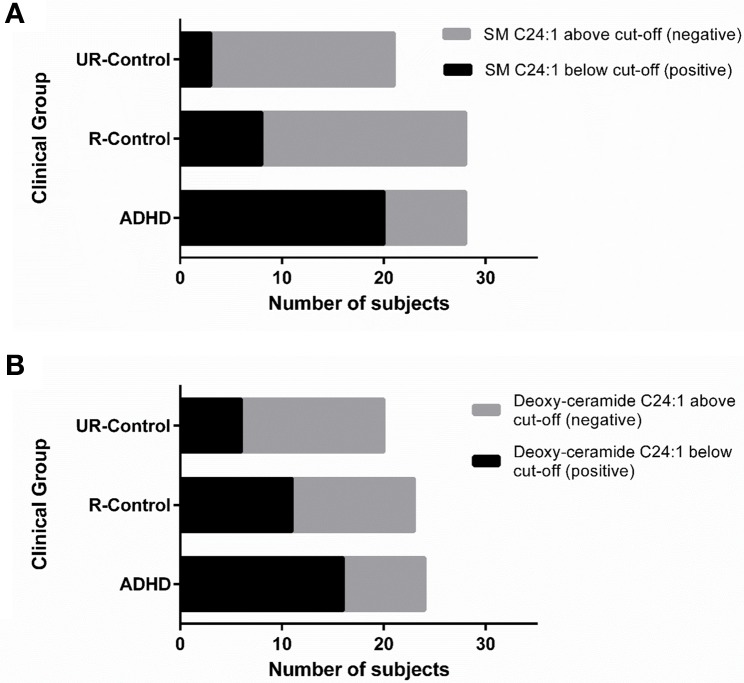
**SM C24:1 and deoxy-ceramide C24:1 may be ADHD endophenotypes**. Contingency graphs showing the number of subjects below and above cutoff among ADHD patients, their unaffected relatives and non/related unaffected controls for **(A)** SM C24:1 and **(B)** deoxy-ceramide C24:1. UR-controls, Unaffected subjects without family history of ADHD; R-Controls, Unaffected relatives of ADHD patients.

To explore whether any of our findings can be considered as a candidate endophenotype (or intermediate phenotype) for ADHD, we evaluated if they would fit an endophenotype-characteristic stair-like distribution, with the highest frequency of the marker among affected subjects, intermediate frequencies among unaffected subjects with positive family antecedents and the lowest frequencies among unaffected subjects without family antecedents of the condition. Based on the ROC curves, we chose the cut-off that best discriminated between ADHD and unaffected subjects for each analyte and built contingency tables that enabled us to evaluate the frequency of subjects below and above the cut-off inside the three clinical groups. The expected stair-like distribution was observed for SM C24:1 and deoxy-ceramide C24:1 (*p*-values: *p* < 0.0001, and *p* = 0.01, respectively) (Figure 5). As a confirmatory approach and in order to adjust significances by age and gender effects, we also adjusted a GLM using the variable “clinical group” as a quantitative variable with three levels, being the highest for ADHD patients and the lowest for non-related controls, with similar results (*p*-values: *p* < 0.001 for SM C24:1 and *p* = 0.03 for deoxy-ceramide C24:1).

## Discussion

In this pilot study, we characterized sphingolipid serum profiles in ADHD patients and two control groups: (1) unaffected first degree relatives of ADHD patients who live and eat with their index ADHD case and (2) unaffected subjects without a family history of ADHD among their first and second degree relatives. Sphingolipids and their metabolites are important for neuronal functioning (Buccoliero and Futerman, 2003; Colombaioni and Garcia-Gil, 2004), but serum concentrations have not been reported in the context of ADHD.

The major finding of our study is that serum levels for all major sphingomyelins and select ceramides are lower in ADHD patients compared to related -as well as to- unrelated controls. The biggest relative difference in sphingomyelin levels among the groups was observed for the long chain sphingomyelin C24:1, with a 21% reduction in the ADHD group when compared to the control groups. It is noteworthy that although more than 100 different subspecies of sphingomyelins have been described in human serum, the four species included in this study account for as much as 60% of total SM, while each one of the remaining non-assayed species account for about 0.5% of total SM (Quehenberger et al., 2010).

Figure 1 shows a schematic representation of sphingolipid metabolic pathways with emphasis on those metabolites that are relevant to our results. Ceramide can originate from *de novo* synthesis from serine and palmitoyl-CoA, or from the recycling pathway through catabolism of complex sphingolipids. Serine-palmitoyl transferase can alternatively utilize alanine instead of serine resulting in the synthesis of deoxyceramides. Degradation of ceramide yields sphingoid bases that can be phosphorylated to sphingosine-1-phosphate. Based on this scheme, potential causes for observed decreased serum sphingomyelins are (1) decreased sphingomyelin uptake or synthesis or (2) increased sphingomyelinase activity. Neither of these mechanisms was specifically evaluated in the current study.

Ceramide C24:0 was decreased 20% and deoxy-ceramide C24:1 -which originates exclusively from the *de-novo* pathway- was decreased 30% in ADHD patients. Notably, the low levels of ceramide C24:0 and deoxy-ceramide C24:1 in ADHD patients are not paralleled by a decrease in any dihydroceramide, suggesting that ceramide synthase-2 activity (which generates dihydroceramides with acyl chains C22–24) might not be impaired in ADHD. Comparable concentrations of all dihydroceramide species also suggest that low ceramide C24:0 and deoxy-ceramide C24:1 are not compensated by increased synthesis of other species through the *de-novo* pathway (see Figure 1). Thus, potential causes for observed decreased ceramides are (1) selectively decreased activity of dihydroceramide desaturase (DES) that converts dihydroceramides to ceramides, or (2) increased conversion of ceramide C24:0 into sphingosine, ceramide-1-phosphate or glucosylceramides, which were not evaluated. Decreased ceramide C24:0 might also be caused by decreased sphingomyelin C24:0, not measured in this study. Such a mechanism, however, cannot explain decreased deoxy-ceramide C24:1, since this ceramide is not a degradation product of sphingomyelin or any other more complex sphingolipids and must be generated *de-novo*. Notably, decreased nervonic acid (C24:1) has been reported in red blood cells of ADHD patients (Chen et al., 2004).

Whether serum sphingolipid profiles reflect brain sphingolipid metabolism is not known. For patients with HIV and Alzheimer's disease, a significant correlation between ceramide and sphingomyelin levels in plasma and CSF have been reported (Mielke et al., 2010, 2011), but no studies have explored these correlations in the general population. Future research is necessary to evaluate if plasma sphingolipid levels can be related to changes in CSF and brain tissue in ADHD.

The interpretation and extrapolation of our results must consider some limitations. First, cases and controls are not age-gender matched and we observe significant differences in the distributions for these variables among the groups (see Table 1). As a consequence, we are not able to completely rule-out a confounding effect for age and gender, even though we controlled by these variables in all subsequent statistical analyses. To date, there is very limited data about physiological changes associated to age and gender in serum sphingolipids. A recent study describes a specific gender effect for the serum levels of several sphingomyelins, with higher values for women (Ishikawa et al., 2014), but we did not observe significant gender effects in anyone of the studied ceramide/sphingomyelin species in our sample (Table 2). The same study did not show age specific effects for any of the sphingolipids that we report to be significantly associated with ADHD. We explored eventual correlations between age and serum sphingolipid levels in our sample and did not find significant correlations for anyone of the analytes included in this study, except for ceramide C24:1, which did not present significant differences between ADHD patients and controls (Table 3). We also performed a subsample analysis considering all participants from 5 to 18 years old (mean age ADHD group = 12.3 years 95% CI = 11.25 − 13.42; mean age controls = 12.2 years 95% CI = 10.87 − 13.7 *p* = 0.72) and were able to confirm our observations in terms of significantly lower levels for all sphingomyelins (SM C16:0 *p* = 0.0028; SM C18:0 *p* = 0.0088; SM C18:1 *p* = 0.018; SM C24:1 *p* = 0.0038). Of note, we also performed ROC analysis for total SM levels using an age-matched subsample, finding sensitivity and specificity values comparable with those reported for the complete sample (AUC_subsample_ = 0.82 CI: 0.66 – 0.93, *p* < 0.0001; 82% sensitivity and 78% specificity), suggesting that results are not affected by age. We also observed the same i.e., decreased albeit non-significant trends for ceramide C24:0 and deoxy-ceramide C24:1 in the sub-sample analyses (C24:1 *F* = 1.1 *p* = 0.3 and deoxy-ceramide C24:1 *F* = 2.36 *p* = 0.14, respectively). As a second limitation, none of our patients were treatment naïve and we cannot rule-out that our findings correspond to a chronic treatment effect instead of being an actual pathogenic marker/factor. Acute treatment effects, on the other hand, were prevented by requiring a 24 h washout period for medication. Finally, our restricted sample size did not allow us to perform analyses by clinical subtype and we are not able to distinguish whether the reported results are applicable to all clinical subgroups or were driven by a specific clinical subtype. We also might be underpowered for detecting some differences of eventual clinical importance. As an example, taking into account the overall variation of our data, we estimate that our study is not able to detect differences less than 20% for the majority of the ceramides. In light of the potential relevance of the results reported by this pilot study, future research addressing these limitations and including the exploration of additional confounders, such as co-morbidities, is mandatory.

**Table 2:** **Gender effects for the studied ceramides and sphingomyelins**.

**Analyte**	***F*-value**	***p*-value**
SM C16:0	0.09	0.77
SM C18:0	0.04	0.85
SM C18:1	1.89	0.18
SM C24:1	0.12	0.73
C16:0	0.01	0.93
C18:0	1.39	0.24
C20:0	2.14	0.13
C22:0	0.35	0.55
C24:0	0.73	0.39
C24:1	0,19	0.67
Deoxy-ceramide C24:1	0.12	0.73

Reported effects are controlled by age and clinical group.

**Table 3:** **Age-serum level correlations for the studied ceramides and sphingomyelins**.

**Analyte**	**Pearson correlation coefficient**	***p*-value**
SM C16:0	0.14	0.91
SM C18:0	−0.02	0.88
SM C18:1	0.07	0.57
SM C24:1	0.15	0.2
C16:0	0.17	0.14
C18:0	0.07	0.54
C20:0	0.14	0.25
C22:0	0.22	0.07
C24:0	0.11	0.38
C24:1	0.25	0.03
Deoxy-ceramide C24:1	0.016	0.89

Reported correlations correspond to partial correlations using gender and presence/absence of ADHD as co-variants.

Strengths of our study include (1) the presence of a comparison group of first degree relatives that live and eat with the index case to control for a potential “diet” effect and for other unknown environmental factors and (2) the inclusion of an additional control group of unaffected subjects without a family history of ADHD to assess replication of findings. This is relevant because differences observed between ADHD patients and their relatives could reveal either a true characteristic of ADHD patients, or reflect compensatory mechanisms exerted by unaffected relatives to constitutional risk factors shared with ADHD patients.

In addition, the experimental design of this study allows to explore whether sphingolipid levels fulfill the distribution criteria proposed by Gottesman and Gould for endophenotypes of psychiatric disorders (Gottesman and Gould, 2003). Endophenotypes are quantifiable markers of genetic liability for non-mendelian complex diseases, which constitute a more “direct” expression of the gene effect than the disorder itself, since they are influenced by fewer genetic and environmental variables. They do not need to be specific, since they might signal common pathogenic mechanisms between related disorders. Our results suggest that sphingomyelin C24:1 and deoxy-ceramide C24:1 qualifies for the expected endophenotype distribution and should be further explored as potential endophenotypes for ADHD. Notably, regions containing genes encoding key enzymes in the sphingolipid metabolism i.e., serine-palmitoyl transferase (SPTLC1, SPTLC3, SPTSSB) and sphingomyelinases (SMPD1, SMPD4, SMPD3A), have been mapped in association with ADHD (Asherson et al., 2008; Romanos et al., 2008; Rommelse et al., 2008).

In conclusion, our results show decreased serum levels of sphingomyelins SM C16:0, SM C18:0, SM C18:1, and SM C24:1, of ceramide C24:0 and deoxy-ceramide C24:1 in ADHD patients compared to related and unrelated controls. This is the first study of serum sphingolipids in ADHD patients. Our data suggest that sphingomyelin SM C24:1 and deoxy-ceramide C24:1 are potential markers for ADHD (endophenotypes) that could reflect pathomechanisms relevant to the disorder.

## Author contributions

Study/Experiments design: MH, SS, and TQ. Experiment execution: MH and BK. Data analysis/Statistical analysis: MH and BK. Interpretation of results and discussion: MH, TW, and RD. Contributed reagents/materials/analysis tools: MH, TW, SS, and TQ. Elaboration of the manuscript: MH and TW. Critic lecture of the manuscript: TW, RD, SS, and TQ.

### Conflict of interest statement

The authors declare that the research was conducted in the absence of any commercial or financial relationships that could be construed as a potential conflict of interest.

## Abbreviations

GLM: general linear model
ROC: receiver operating characteristic
SM: sphingomyelin.
